# Potential for reduced radiation‐induced toxicity using intensity‐modulated arc therapy for whole‐brain radiotherapy with hippocampal sparing

**DOI:** 10.1120/jacmp.v16i5.5587

**Published:** 2015-09-08

**Authors:** Damodar Pokhrel, Sumit Sood, Christopher Lominska, Parvesh Kumar, Rajeev Badkul, Hongyu Jiang, Fen Wang

**Affiliations:** ^1^ Department of Radiation Oncology The University of Kansas Cancer Center Kansas City KS USA

**Keywords:** hippocampal sparing, whole brain, IMAT, OARs, radiation toxicity

## Abstract

The purpose of this study was to retrospectively investigate the accuracy, plan quality, and efficiency of using intensity‐modulated arc therapy (IMAT) for whole brain radiotherapy (WBRT) patients with sparing not only the hippocampus (following RTOG 0933 compliance criteria) but also other organs at risk (OARs). A total of 10 patients previously treated with nonconformal opposed laterals whole‐brain radiotherapy (NC‐WBRT) were retrospectively replanned for hippocampal sparing using IMAT treatment planning. The hippocampus was volumetrically contoured on fused diagnostic T1‐weighted MRI with planning CT images and hippocampus avoidance zone (HAZ) was generated using a 5 mm uniform margin around the hippocampus. Both hippocampi were defined as one paired organ. Whole brain tissue minus HAZ was defined as the whole‐brain planning target volume (WB‐PTV). Highly conformal IMAT plans were generated in the Eclipse treatment planning system for Novalis TX linear accelerator consisting of high‐definition multileaf collimators (HD‐MLCs: 2.5 mm leaf width at isocenter) and 6 MV beam for a prescription dose of 30 Gy in 10 fractions following RTOG 0933 dosimetric criteria. Two full coplanar arcs with orbits avoidance sectors were used. In addition to RTOG criteria, doses to other organs at risk (OARs), such as parotid glands, cochlea, external/middle ear canals, skin, scalp, optic pathways, brainstem, and eyes/lens, were also evaluated. Subsequently, dose delivery efficiency and accuracy of each IMAT plan was assessed by delivering quality assurance (QA) plans with a MapCHECK device, recording actual beam‐on time and measuring planed vs. measured dose agreement using a gamma index. On IMAT plans, following RTOG 0933 dosimetric criteria, the maximum dose to WB‐PTV, mean WB‐PTV D2%, and mean WB‐PTV D98% were 34.9±0.3 Gy,33.2±0.4 Gy, and 26.0±0.4 Gy, respectively. Accordingly, WB‐PTV received the prescription dose of 30 Gy and mean V30 was 90.5%±0.5%. The D100%, and mean and maximum doses to hippocampus were 8.4±0.3 Gy,11.2±0.3 Gy, and 15.6±0.4 Gy, on average, respectively. The mean values of homogeneity index (HI) and conformity index (CI) were 0.23×0.02 and 0.96×0.02, respectively. The maximum point dose to WB‐PTV was 35.3 Gy, well below the optic pathway tolerance of 37.5 Gy. In addition, compared to NC‐WBRT, dose reduction of mean and maximum of parotid glands from IMAT were 65% and 50%, respectively. Ear canals mean and maximum doses were reduced by 26% and 12%, and mean and maximum scalp doses were reduced by 9 Gy (32%) and 2 Gy (6%), on average, respectively. The mean dose to skin was 9.7 Gy with IMAT plans compared to 16 Gy with conventional NC‐WBRT, demonstrating that absolute reduction of skin dose by a factor of 2. The mean values of the total number of monitor units (MUs) and actual beam on time were 719×44 and 2.34×0.14 min, respectively. The accuracy of IMAT QA plan delivery was (98.1±0.8) %, on average, with a 3%/3 mm gamma index passing rate criteria. All of these plans were considered clinically acceptable per RTOG 0933 criteria. IMAT planning provided highly conformal and homogenous plan with a fast and effective treatment option for WBRT patients, sparing not only hippocampi but also other OARs, which could potentially result in an additional improvement of the quality life (QoL). In the future, we plan to evaluate the clinical potential of IMAT planning and treatment option with hippocampal and other OARs avoidance in our patient's cohort and asses the QoL of the WBRT patients, as well as simultaneous integrated boost (SIB) for the brain metastases diseases.

PACS number: 87

## I. INTRODUCTION

Whole‐brain radiotherapy (WBRT) is a common treatment modality in patients with multiple brain metastases,^(1)^ given as prophylactic cranial irradiation (PCI) for non‐small‐cell^(2)^/small‐cell lung cancer,^3^, ^4^ and cranial and craniospinal irradiation for pediatric/adult central nervous system malignancies.^5^, ^6^ For solitary brain metastasis postoperatively, WBRT reduced intracranial failure from 70% to 18%, an absolute gain of 52% in any brain failure compared to without WBRT.^(1)^ The use of PCI significantly reduced the obvious development of brain metastases at one year 7.7% vs. 18% for PCI vs. observation without improved overall survival benefit reported by RTOG 0214 phase III non‐small‐cell lung cancer study.^(2)^ For limited stage small‐cell lung cancer, three years' overall survival improved from 14% to 21%, and decreased incidence of brain metastases from 59% to 33% due to PCI.^(3)^ Similarly, the use of PCI for extensive stage small‐cell lung cancer has shown three years' overall survival improved from 13% to 27% and reduced incidence of brain metastases from 40% to 15% at one year.^(4)^ Sixty‐seven percent of craniospinal pediatric patients have shown five‐year disease‐free survival rates after craniospinal irradiation (CSI)^(5)^ and 55% of adult craniospinal patients survive more than three years after CSI.^(6)^ However, clinical and preclinical evidence suggest that irradiating a neural stem cell compartment in the hippocampus introduces radiation‐induced neurocognitive toxicity/deficits (short‐ and long‐term memory loss) after conventional nonconformal WBRT within the first one to four months, leading to compromise in QoL.^7^, ^8^, ^9^

To address this issue, researchers have developed helical tomotherapy^(10^, ^11)^ or linear accelerator‐based, intensity‐modulated radiation therapy (IMRT) techniques^(11^, ^12)^ with hippocampal sparing that could conformally avoid the hippocampi and significantly reduce the amount of radiation dose to the neural stem cell compartment in the hippocampus. However, tomotherapy or linear accelerator‐based IMRT requires a large number of total MUs and relatively longer treatment times.^10^, ^11^, ^12^

A recently introduced rotational radiotherapy technique, intensity‐modulated arc therapy (IMAT) treatment planning system, delivers a highly conformal radiation dose to the target by simultaneously modulating gantry rotation, dose rate, and multileaf collimators (MLCs) position.^13^, ^14^, ^15^, ^16^, ^17^ The advantages of the IMAT system over IMRT include the ability to reduce the total number of MUs and subsequently the beam‐on time, which may improve patient tolerance of treatment, and potentially reduce leakage radiation dose to the patients.^13^, ^14^ Conformal IMAT plans may decrease the dose to the organs at risk (OARs), including the hippocampus. In this report, we present a feasibility study to explore the clinical potential for IMAT for the fast and effective delivery of WBRT with hippocampal sparing following RTOG 0933 dosimetric compliance criteria.^18^, ^19^ We simultaneously evaluate our ability to spare other non‐target organs which are treated in traditional NC‐WBRT fields, including the scalp, ear canals, cochleae, skin, and parotid glands.

## II. MATERIALS AND METHODS

### A. Patient simulation and target volume definition

A total of 10 patients, who were diagnosed with stage IV small‐cell/non‐small‐cell lung cancer and who underwent whole‐brain radiation therapy for brain metastasis using nonconformal opposed lateral fields at the University of Kansas Hospital, Kansas City, were included in this retrospective study. During the computed tomography (CT) simulation scan, patients were immobilized in a supine position on a 16 slice Phillips Brilliance Big Bore CT Scanner (Phillips Healthcare, Andover, MA) using arms on the chest with blue ring and knee roll. The 3D CT images were acquired with 512×512 pixels at 2.5 mm slice thickness and 2.5 mm slice spacing. At the simulation CT, a staff radiation oncologist marked the isocenter at the middle of the whole brain. The DICOM images with the isocenter marked were then electronically transferred to the Eclipse treatment planning system (Varian Medical Systems, Palo Alto, CA) for WBRT planning. Whole‐brain tissue and right and left eyes/lenses were then automatically contoured in the Eclipse treatment planning system using model‐based segmentation for the conventional nonconformal whole‐brain irradiation.

### B. Conventional NC‐WBRT treatment planning

In our clinic, all of these patients were planned in Eclipse TPS (version 10.0.28) with analytical anisotropic algorithm (AAA) using conventional NC‐WBRT technique with parallel opposed lateral portals and treated with the Novalis‐TX linear accelerator (Varian Medical Systems) and 6 MV beam arrangement. The parallel opposed lateral NC‐WBRT field apertures were generated using open field that included the whole‐brain parenchyma. Inferior field border was placed inferior to the cribriform plate, the middle cranial fossa, and the foramen magnum. Approximately 1 cm safety margins were subsequently generated to account for penumbra width, head fixation, and other anatomic factors. The anterior border of the field was placed approximately 2 to 3 cm posterior to the ipsilateral eyelid to prevent beam divergence into the contralateral lens. High‐definition‐MLCs (2.5 mm leaf width at isocenter) were used to create the field apertures described. The prescription dose was total 30 Gy in 10 fractions at the isocenter.

### C. Whole‐brain IMAT treatment planning

After obtaining institutional review board (IRB) approval from the University of Kansas Cancer Center, Kansas City, KS, all those clinical NC‐WBRT plans were retrieved and replanned for the retrospective IMAT planning study. The T1‐weighted cranial magnetic resonance imaging (MRI) scan was rigidly registered to the bony anatomy on the planning CT images by using an Eclipse mutual information algorithm. The hippocampi, optic nerves, optic chiasm, and brain stem were manually delineated by an experienced radiation oncologist on the T1‐weighted MRI images and mapped on the planning CT images for dose planning and reporting. Hippocampal avoidance zone (HAZ) was defined as the hippocampus plus uniform 5 mm margin around the hippocampus as a planning structure for the dose reduction/optimization. The whole‐brain planning target volume (WB‐PTV) was defined as whole‐brain tissue minus HAZ, following RTOG 0933 guidelines.^(18)^ In addition, other structures, including the parotid glands, scalp, skin, external/middle ear canals, and cochlea, were delineated for dose reporting.

**Figure 1:**
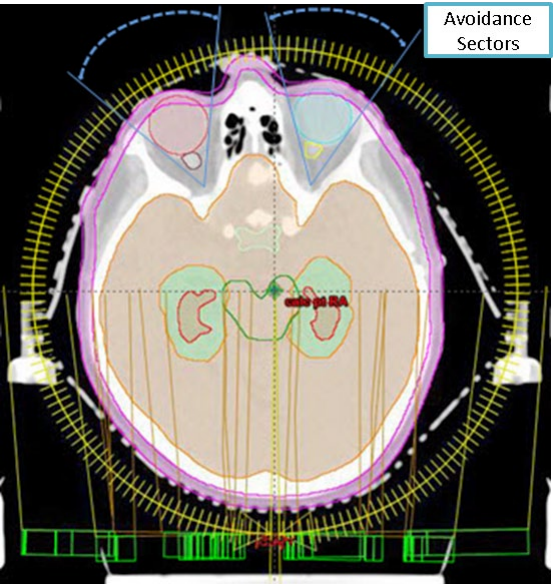
Demonstration of the transverse view of IMAT planning setup (two full coplanar arcs with bilateral orbital exclusion sectors) in the Eclipse TPS for hippocampal‐sparing WBRT (Patient # III) with respect to patient anatomy. Red shaded region represents the hippocampus and light‐blue contour represents the HAZ.

For all IMAT plans, 6 MV beam for the Novalis‐TX linear accelerator equipped with HD‐MLC (2.5 mm leaf width at isocenter) was used at a maximum dose rate of 600 MUs/min. The isocenter was kept at the center of the whole‐brain tissue, the same as in conventional NC‐WBRT plans based on beam's eye view graphic. Two full coplanar arcs covered 358° gantry rotation. The clockwise arc used a 30° collimator rotation, with the counterclockwise arc at a complementary 330° collimator rotation to curtail the MLC tongue‐and‐groove leaves' leakage for the IMAT plans. Bilateral orbits avoidance sectors were introduced in the IMAT plans for at least 20° gantry angles for each arc to avoid direct beam entrance through the eyes. All treatment plans were calculated using anisotropic analytical algorithm (AAA) for heterogeneity corrections (Varian Eclipse TPS version 10.0.28) with $2.5\times 2.5\times 2.5\;{\text{mm}}^{3}$ dose grid sizes. All plans had a dose delivery schema of 30 Gy in 10 fractions, with at least 98% of the WB‐PTV receiving 25 Gy and 90% of WB‐PTV receiving the prescription dose (i.e., D90%=30 Gy). Even though RTOG 0933 allowed D2% less than 37.5 Gy to the WB‐PTV (see Table 1), these IMAT plans were optimized to keep the maximum point dose to the WB‐PTV under 35.3 Gy. In order to achieve better WB‐PTV coverage and lower OARs dose tolerances, all the IMAT plans were inversely optimized using variation of the multileaf collimator's (MLC) leaf positions, gantry rotation speed, and dose rate, following RTOG 0933 dosimetric compliance criteria.^(18)^ Fig. 1 shows the IMAT treatment planning setup.

**Table 1:** RTOG 0933 dosimetric compliance criteria for hippocampal sparing

*Organ*	*Dose Constraints*
Whole Brain PTV	D2%<37.5 Gy(D2%<40 Gy is allowed) D98%>25 Gy andV30>90%
Hippocampus	D100%<9 Gy(D100%<10 Gy is allowed) Dmax<16 Gy(Dmax<17 Gy is allowed)
Maximum dose to optic chiasm and optic nerves	<37.5 Gy

### D. Whole‐brain IMAT treatment plan evaluation

The DVHs of all whole‐brain IMAT treatment plans were generated in the Eclipse TPS for the WB‐PTV, hippocampus, and optic pathway. All plans were evaluated following RTOG 0933 dosimetric compliance criteria (see Table 1 for the RTOG criteria).^(18)^

Also, dosimetric evaluation of these plans was performed by calculating CI and HI using the DVHs of the WB‐PTV for all IMAT plans.

The CI was defined as follows: ^(20)^

(1) $$CI=\frac{V_{ip}}{V_{\left(WB-PTV\right)}}$$

where $V_{ip}$ represents the treated volume enclosed by the prescription isodose line and $V_{\left(WB-PTV\right)}$ represent the WB‐PTV. The CI values closest to unity indicate superior plan conformity of dose distributions to the WB‐PTV. The HI index was computed using the following formula: ^(21)^

(2) $$HI=\frac{\left(D2\%-D98\%\right)}{D_{median}}$$

where D2% and D98% correspond to the dose given to 2% and 98% of the WB‐PTV, respectively; Dmedian was the median dose to the WB‐PTV. Smaller values of HI indicate better dose homogeneity within the WB‐PTV.

### E. Radiation dose to hippocampus

Following the RTOG 0933 dosimetric compliance criteria, the maximum dose and dose to 100% of hippocampus volume (D100%) were documented for all IMAT plans and compared with conventional NC‐WBRT treatment. Statistical analysis was performed using Microsoft Excel (Microsoft Corp, Redmond, WA) program. Mean and standard deviation (SD) values for each of the dose metrics were compared using two‐tailed paired *t*‐tests between the hippocampal‐sparing IMAT vs. conventional NC‐WBRT plans using an upper bound of p‐value <0.001.

### F. Radiation dose to other OARs

In addition to RTOG 0933 parameters analysis, all the IMAT plans were evaluated for the other OARs doses, such as mean doses to parotid glands, scalp, skin, and cochlea, as well as the percent of the ear canals receiving 30 Gy (V30%). The maximum doses to eyes/lenses were also documented and compared against conventional NC‐WBRT plans.

### G. IMAT dose delivery efficacy and accuracy

Dose delivery efficiency was evaluated based on reporting total number of MUs and actual beam‐on time required to deliver the given prescription dose for all IMAT plans. For each plan, actual beam‐on time was recorded at the treatment machine while delivering IMAT QA plan. Delivery accuracy of the IMAT QA plan was assessed by physically measuring the 2D dose distributions for all plans in an in‐house static plastic phantom which housed the MapCHECK (Sun Nuclear Cop., Melbourne, FL) device. The plastic phantom was $30\times 30\times 20\;{\text{mm}}^{3}$ in dimension, providing buildup of 10 cm at the top and bottom as well as 5 cm on all other sides. All the QA plans were delivered at the machine in one session, minimizing dependence of the IMAT QA result on machine output rate. The measured cumulative 2D dose plan was compared with the computed dose distributions calculated on the MapCHECK QA phantom by the Eclipse treatment planning system. Upon completion of measurements, data were analyzed with MapCHECK software (SNC patient, version 6.1) using the clinical gamma passing rate criteria of 3% maximum dose difference and 3 mm distance to agreement (DTA) with 10% threshold.

## III. RESULTS

**Figure 2:**
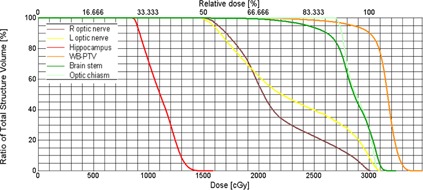
A DVH for hippocampal‐sparing WBRT using IMAT planning (Patient # III). A dose of 30 Gy in 10 fractions was prescribed to achieve better than 90% of the WB‐PTV coverage. In this case, maximum dose to WB‐PTV, D2%, D98%, and $D_{\text{median}}$ was 34.8 Gy, 33.2 Gy, 26.1 Gy, and 31.7 Gy, respectively. Maximum and D100% dose to hippocampus were <16 Gy and <9 Gy, respectively. Optic pathway and brainstem doses were all well below RTOG 0933 guidelines.

**Figure 3:**
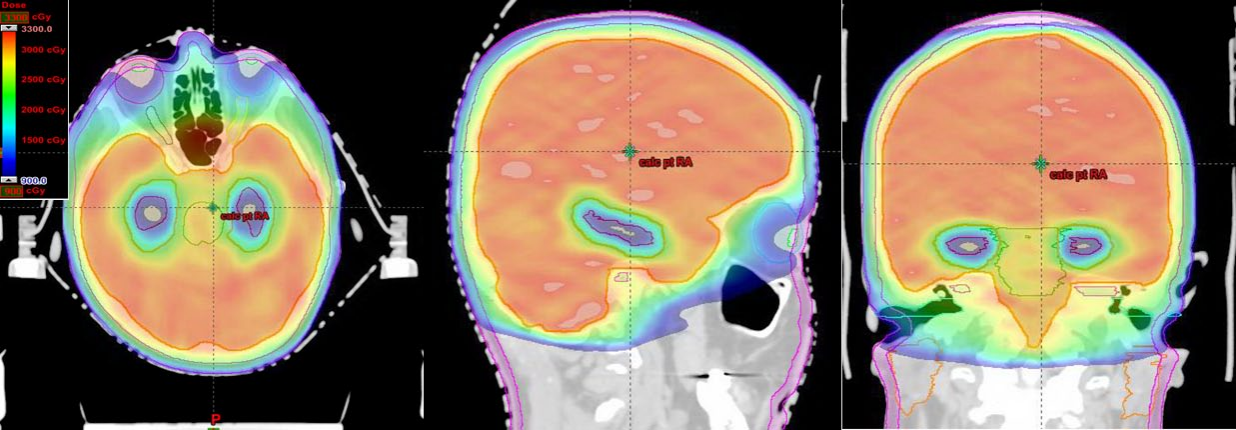
Spatial dose distributions in axial, sagittal, and coronal views for one representative hippocampal‐sparing WBRT patient (Patient # III) using IMAT treatment planning. Red contoured region represents the hippocampus. Light‐blue contour represents the HAZ. Blue isodose colorwash represents 9 Gy; light blue, 15 Gy; light green, 25 Gy; red, 30 Gy; orange, 33 Gy, in 10 fractions. Other normal tissues structures such as parotid glands (orange contour), skin (pink contour), and brain stem (green contour) are clearly shown.

An IMAT‐computed DVH for one representative hippocampal‐sparing WBRT patient (Patient # III) is shown in Fig. 2. The dose distribution in axial, coronal and sagittal views for the same representative patient is shown in Fig. 3.

2, 3 present the results of dosimetric parameters for 10 hippocampal‐sparing whole‐brain IMAT plans corresponding to RTOG 0933 dosimetry evaluation criteria. The WB‐PTV maximum, D2%, and D98% doses were 34.9±0.3 Gy (range 34.4 to 35.3 Gy); 33.2±0.4 Gy (range 32.8 to 33.9 Gy); and 26.0±0.4 Gy (range 25.5 to 26.9 Gy), on average, respectively. Since the highest maximum point dose to the WB‐PTV was 35.3 Gy (Patient # V), for all patients' plans the maximum doses to the optic nerves, optic chiasm, and brainstem were all below RTOG guidelines. The average value of the WB‐PTV receiving the prescription dose of 30 Gy (V30) was 90.5%±0.5% (range 90.1% to 91.5%). The mean values of HI and CI were 0.23±0.02 (range 0.19 to 0.24), and 0.96±0.02 (range 0.94 to 1.01), respectively, demonstrating highly conformal and homogenous plans. The maximum dose and dose to 100% of hippocampus (D100%) were 15.6±0.3 Gy (range 15.2 to 15.9 Gy), and 8.4±0.3 Gy (range 8.1 to 8.7 Gy), on average, respectively. The mean value of hippocampus dose for this group of patients was 11.2±0.3 Gy (see Table 3). Hippocampus maximum dose on average was 15.6±0.4 Gy. The average value of median dose for this group of patients with NC‐WBRT was 31.7±0.2 Gy, similar, to that of HC sparing IMAT plans.

**Table 2:** The WB‐PTV coverage, HI, and CI from WBRT with hippocampal sparing using IMAT technique. The WB‐PTV coverage for all 10 WBRT patients was compliant with RTOG 0933 requirement

*Patient #*	*WB‐PTV max (Gy)*	*WB‐PTV D2% (Gy)*	*WB‐PTV D98% (Gy)*	*WB‐PTV* $D_{\text{median}}$ *(Gy)*	*WB‐PTV V30 (%)*	*HI*	*CI*
I	34.6	32.9	25.7	31.6	90.1	0.23	0.95
II	35.0	33.2	25.7	31.8	90.2	0.24	0.97
III	34.8	33.2	26.1	31.7	90.3	0.22	0.94
IV	34.5	32.8	26.9	31.3	91.5	0.19	0.94
V	35.3	33.8	26.1	32.6	90.1	0.23	1.01
VI	34.7	32.8	26.4	31.3	91.0	0.20	0.95
VII	35.2	33.5	25.5	31.7	90.1	0.25	0.96
VIII	34.4	32.9	26.2	31.6	90.9	0.21	0.96
IX	35.2	33.6	25.9	32.1	90.1	0.24	0.97
X	34.8	33.3	25.7	31.8	90.2	0.24	0.94
AVG	34.9	33.2	26.0	31.7	90.5	0.23	0.96
STDEV	0.3	0.4	0.4	0.4	0.5	0.02	0.02

AVG=average,STDEV=standard deviation.

**Table 3:** The dose to hippocampus (HC) from WBRT with hippocampal sparing using IMAT technique. The hippocampi doses for all 10 WBRT patients met the RTOG 0933 requirement

*Patient #*	*HC* D100% *(Gy)*	*HC max (Gy)*	*HC mean (Gy)*
I	8.5	15.5	11.1
II	8.4	15.9	11.3
III	8.5	15.8	11.1
IV	8.1	15.7	11.1
V	8.8	15.9	11.7
VI	8.0	14.8	10.8
VII	8.2	15.7	11.2
VIII	8.4	15.2	11.3
IX	8.7	15.8	11.4
X	8.4	15.8	11.4
AVG	8.4	15.6	11.2
STDEV	0.3	0.4	0.3

HC=hippocampus,AVG=average,STDEV=standard deviation.

**Figure 4:**
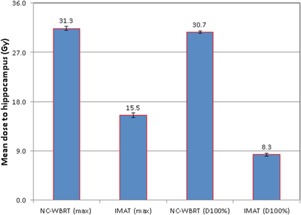
Bar graphs with associated SD error bars comparing the average (total 10 patients) maximum dose and dose to 100% of hippocampus (D100%) delivered using conventional NC‐WBRT vs. hippocampal‐sparing IMAT planning. IMAT treatment planning technique significantly reduced the both maximum dose (mean value <15.5±0.4 Gy) and D100% to hippocampus (mean value <8.3±0.3 Gy), in accordance with RTOG 0933 requirement.

Following the RTOG guidelines, hippocampal‐sparing IMAT treatment planning technique was able to reduce maximum dose by almost half and D100% to hippocampus by better than threefold (see Fig. 4) for those patients' plans who were treated with conventional NC‐WBRT (p‐value <0.001, student *t*‐test) without compromising the WB‐PTV coverage.

Table 4 presents detailed information on total number of MUs, beam‐on time, and QA pass‐rate values for all 10 WBRT IMAT plans. The total number of MUs was 719±44, on average, and ranged from 636 to 791. The average beam‐on time was 2.34±0.14 min. The dose delivery accuracy of these IMAT plans was 98.1%±0.8%, on average, with 3%/3 mm clinical gamma passing rate criteria.

In addition to the hippocampal sparing, IMAT plans significantly reduced doses to other OARs such as parotid glands, external/middle ear canals, skin, scalp, eyes/lenses, and cochleae, compared to NC‐WBRT. In summary, the following is the absolute reduction in OARs doses as a by‐product of hippocampal sparing for WBRT: the average values of mean dose received by left and right parotid glands reduced from 14.5 Gy to 4.2 Gy (p‐value <0.001), and 13.0 Gy to 4.6 Gy (p‐value <0.001), respectively, when compared to NC‐WBRT plans. The percent of left and right ear canals receiving 30 Gy dose was 0% vs. 52.1% (p‐value <0.001) and 0% vs. 51.7% (p‐value <0.001), on average, for IMAT vs. conventional NC‐WBRT plans, indicating that none of the ear canals even received 30 Gy point dose with IMAT plans. The mean values of maximum dose received by left and right eyes was reduced from 31.9 Gy to 17.6 Gy (p‐value <0.001), and 31.8 Gy to 16.6 Gy (p‐value <0.001), respectively, compared to conventional NC‐WBRT plans, showing that maximum eyes doses reduced by almost half with IMAT plans. The mean dose to scalp was less than 19 Gy with IMAT plans vs. 28 Gy (p‐value <0.001) with conventional NC‐WBRT. Similarly, mean dose to skin was 9.7 Gy with IMAT plans vs. 16 Gy (p‐value <0.001) with conventional NC‐WBRT, demonstrating that absolute reduction of skin dose by a factor of 2. Also, the mean dose to both left and right cochlea was reduced by about 4 Gy each, on average, while providing similar maximum dose to the left/right lens, on average, compared to conventional NC‐WBRT.

**Table 4:** Detailed information on total number of MUs, beam‐on time, and IMAT QA pass rate values for all 10 WBRT patients using IMAT planning

*Patient #*	*Total # of MUs*	*Beam‐on Time (min)*	*Gamma Pass Rate* 3%/3 mm *(%)*
I	763	2.48	98.8
II	714	2.32	97.5
III	712	2.31	99.1
IV	749	2.43	97.7
V	638	2.07	98.6
VI	791	2.57	97.4
VII	725	2.36	96.9
VIII	689	2.24	97.5
IX	680	2.21	99.2
X	730	2.37	97.8
AVG	719	2.34	98.1
STDEV	44	0.14	0.8

MUs=monitor units,AVG=average,STDEV=standard deviation.

## IV. DISCUSSION

In this study, we have presented the feasibility of fast and effective treatment planning and delivery for WBRT using IMAT technique with hippocampal sparing following RTOG criteria. Providing excellent conformity and homogeneity indices, hippocampal‐sparing IMAT planning could potentially reduce radiation‐induced inflammation to hippocampus, and its associated neurologic functional sequelae, without compromising target coverage. All of our IMAT plans met all RTOG 0933 dosimetric compliance requirements. Using helical tomotherapy and linac‐based IMRT technique, Gondi and colleagues ^(11)^ have presented excellent results in sparing the hippocampi in the course of WBRT treatments for patients with brain metastasis. For five WBRT patients, with helical tomotherapy the median dose to the hippocampus was 5.5 Gy and maximum dose of 12.8 Gy. Linac‐based noncoplanar IMRT spared the hippocampus, with a median dose of 7.8 Gy and maximum dose of 15.3 Gy. However, no treatment time was reported. Another study presented by Nevelsky et al.^(12)^ used Elekta equipment with nine noncoplanar IMRT beams and Monaco TPS to spare hippocampus. Following the RTOG guidelines, in 10 patient plans, Nevelsky and colleagues have shown dose to 100% of hippocampus 8.4 Gy, on average, and the hippocampus maximum dose mean value 14.4 Gy with an excellent IMRT QA results. However, their mean number of total MUs was 1724 and, consequently, they had longer treatment times of about 12 min.

Our IMAT treatment planning study uses two full coplanar arcs with bilateral orbits avoidance sectors. Our mean dose to hippocampus was 8.4 Gy and maximum 15.6 Gy without compromising the WB‐PTV coverage according to the RTOG guidelines. This translated into significant reduction of hippocampal dose compared to conventional NC‐WBRT (p‐value <0.001). IMAT plans were highly conformal and homogeneous, demonstrating that IMAT can deliver conformal dose distributions similar to those found in tomotherapy or linac‐based IMRT treatment plans, allowing for potential expansion of conformal techniques to palliative cases without additional patient setup effort. Our beam on time was 2.3 min, and IMAT QA gamma passing rate was 98.1% (for 3%/3 mm DTA criteria), on average, showing excellent clinical potential for fast and reliable treatment options for WBRT patients with reduced radiation induced toxicity.

Another potential clinical application of IMAT treatment planning and delivery is concurrent boost dose for the brain metastases while simultaneously treating WBRT. Using volumetric‐modulated arc therapy (VMAT) planning, simultaneous integrated boost (SIB) dose delivery has been studied by several researchers^10^, ^22^, ^23^, ^24^, ^25^, ^26^ using varieties of prescription doses in the treatment of up to eight brain tumors. Specifically, Gutierrez and colleagues ^(10)^ evaluated the feasibility of using helical tomotherapy for WBRT with hippocampal sparing and SIB to the brain metastases. For 10 patients, the whole‐brain dose was prescribed 32.5 Gy in 15 fractions and SIB dose to individual brain tumors ranged from 63 Gy to 70.8 Gy. In their study, the hippocampus was conformally avoided with the mean normalized dose to about 6 Gy, and treatment times were reported from about 10 to 22 min. Similarly, Prokic and colleagues ^(24)^ developed a new treatment planning strategy in WBRT patients with hippocampal sparing with stereotactic SIB or sequential boost (SB) concepts. The VMAT plans were generated for 10 patients with up to eight brain tumors for 30 Gy in 12 fraction to the whole brain and 51 Gy in 12 fractions to individual brain tumors (in SIB) and 30 Gy in 12 fraction to the whole brain and 18 Gy in 2 fractions to brain metastases (in SB) with 5 mm and 10 mm HAZ. Both planning techniques were able to achieve adequate whole‐brain coverage and radiosurgical quality of dose distributions to each of the brain metastases and spared hippocampi. A study by Awad et al.^(26)^ presented clinical data on hippocampus sparing for WBRT patients using VMAT with SIB for brain metastases from primarily melanoma origin. In their institution, 30 patients with 73 brain tumors were treated with VMAT. The median whole‐brain dose was 31 Gy with median SIB dose to brain metastases of 50 Gy in 15 fractions. Mean and maximum hippocampus dose was 20.4 Gy and 32.4 Gy, respectively, for patients treated with hippocampal‐sparing technique. The average VMAT treatment time was about 3.4 min and the median overall survival was about 9.4 months. All these peer‐reviewed articles document clinical potential (fast and effective delivery) of SIB treatment to WBRT patients using VMAT planning and also spare hippocampus.

One potential concern for hippocampal‐sparing WBRT is loss of metastatic tumor control near the hippocampus. This concern has been addressed by at least two publications. Gondi et al.^(27)^ performed a comprehensive multi‐institutional study by reviewing 371 patients with 1133 brain tumors with HAZ consisting of the hippocampi plus a 5 mm margin using clinical and radiographic variables. Their study revealed that no patients had metastases within the hippocampus, and the incidence of brain metastases within 5 mm of the hippocampus was about 8.6%. Another study by Chang et al.^(28)^ used the distance from the hippocampus at which the parenchyma would receive less than specified dose, and generated a mathematical model using published data to predict the incidence of potential brain metastases being underdosed for different levels of hippocampal sparing using helical tomotherapy and VMAT plans. Chang and colleagues recommended keeping the mean hippocampus dose below 12 Gy out of 30 Gy in 10 fractions prescription. Their decision‐making guidelines also favor the benefit of decreased dose to hippocampi when deciding on hippocampal‐sparing WBRT treatment. In‐depth clinical rational, feasibility studies for hippocampal‐sparing WBRT including concurrent boost to brain metastatic diseases using modern radiotherapy systems, controversies, and future directions for hippocampal avoidance in cranial irradiation have also been discussed in a review article published by Kazda and colleagues.^(29)^

Radiation‐induced toxicity for WBRT patients has also been reported for other OARs such as parotid glands,^30^, ^31^ scalp,^(32)^ and ear canals.^(33)^ Radiation‐induced toxicity to parotid glands is associated with xerostomia or dry mouth, impaired swallowing, and malnutrition, and unwanted scalp dose with hair loss. Volume of ear canals tissue receiving 30 Gy dose is associated with acute otitis. ^(33)^ Our IMAT plan spared these critical structures without compromising WB‐PTV coverage. Reduction in clinically significant dose to critical structures may improve patient QoL. Detailed explanation of the OARs doses analysis showing the clinical potential of the IMAT planning for reduced radiation‐induced normal tissues toxicity and its clinical significant will be presented in another paper.

In our study, we have demonstrated the feasibility of using IMAT treatment planning and delivery technique to not only satisfy the RTOG 0933 criteria for hippocampal sparing, but also to reduce the normal tissue toxicity for other OARs such as parotid glands, ear canals, scalp, and skin. We believe that reducing hippocampus doses, as well as doses to other OARs, could provide better QoL for those groups of WBRT patients who exhibit longer survival and/or receive PCI, or for pediatric or younger adult patients treated with craniospinal irradiation. Fast and effective delivery of SIB for the brain tumors with WBRT using single or multiple arcs merits further investigation.

## V. CONCLUSIONS

The dosimetric results for our hippocampal‐sparing WBRT treatment planning study using IMAT technique indicate that all plans met clinically acceptable dosimetric compliance criteria set by RTOG 0933. IMAT planning provided highly conformal and homogenous plans, as well as fast and accurate treatment for WBRT, with potential for reduced radiation‐induced toxicity. Normal tissue sparing, including hippocampal sparing, was evident versus NC‐WBRT. We demonstrate sparing of additional non‐target structures including parotid glands, scalp, ear canals, skin, cochlea, and eyes/lenses. IMAT treatment planning and delivery has the clinical potential for improving patient comfort, significantly reducing normal tissue toxicity including hippocampi and other OARs, and incorporating SIB treatment for the metastatic brain tumors.

## Supporting information

Supplementary Material

Supplementary Material

Supplementary Material
