# Supplementary figures and images for: Potential for reduced radiation‐induced toxicity using intensity‐modulated arc therapy for whole‐brain radiotherapy with hippocampal sparing
Source: J Appl Clin Med Phys. 2015 Sep 8;16(5):131–41. doi: 10.1120/jacmp.v16i5.5587 (PMC5690185; doi:10.1120/jacmp.v16i5.5587)

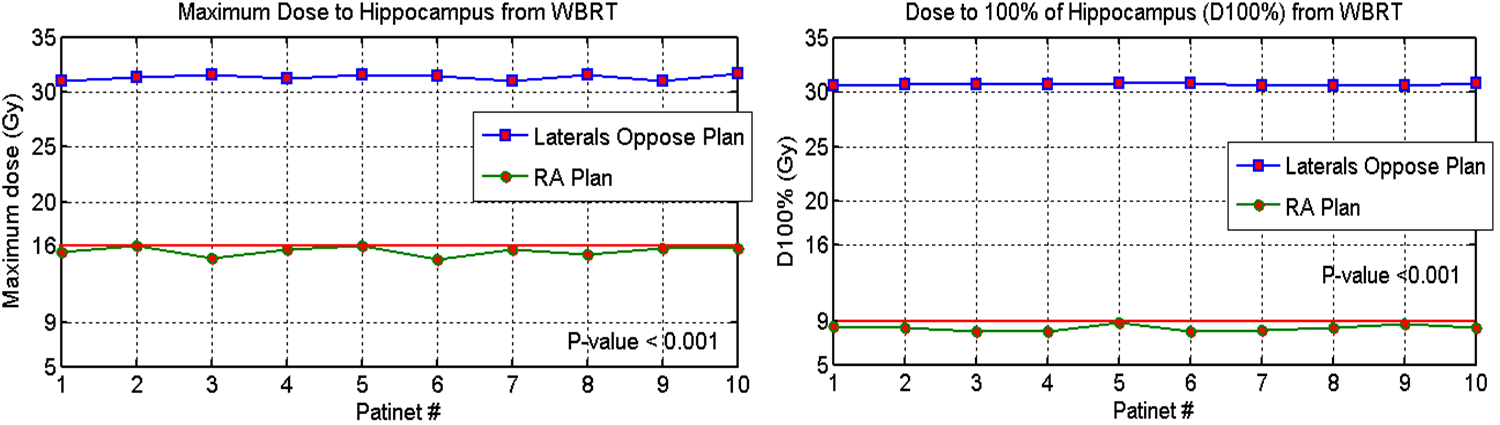

Supplement: Supplementary file 1 — Supplementary Material [file ACM2-16-131-s001.png]

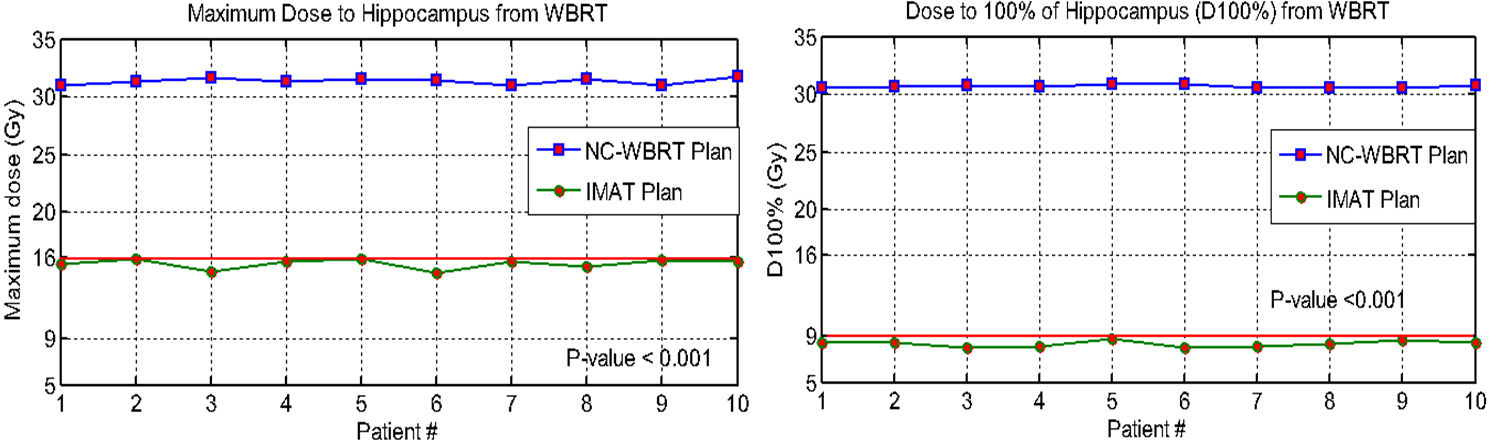

Supplement: Supplementary file 2 — Supplementary Material [file ACM2-16-131-s002.png]
